# Visualizing Sweetness: Increasingly Diverse Applications for Fluorescent-Tagged Glucose Bioprobes and Their Recent Structural Modifications

**DOI:** 10.3390/s120405005

**Published:** 2012-04-18

**Authors:** Woong Hee Kim, Jinho Lee, Da-Woon Jung, Darren R. Williams

**Affiliations:** New Drug Targets Laboratory, School of Life Sciences, Gwangju Institute of Science and Technology, Gwangju 500-712, Korea; E-Mails: kuhhan@hanmail.net (W.H.K.); jh0803@nate.com (J.L.)

**Keywords:** glucose homeostasis, fluorescent-tagged glucose, bioprobe, bioimaging

## Abstract

Glucose homeostasis is a fundamental aspect of life and its dysregulation is associated with important diseases, such as cancer and diabetes. Traditionally, glucose radioisotopes have been used to monitor glucose utilization in biological systems. Fluorescent-tagged glucose analogues were initially developed in the 1980s, but it is only in the past decade that their use as a glucose sensor has increased significantly. These analogues were developed for monitoring glucose uptake in blood cells, but their recent applications include tracking glucose uptake by tumor cells and imaging brain cell metabolism. This review outlines the development of fluorescent-tagged glucose analogues, describes their recent structural modifications and discusses their increasingly diverse biological applications.

## 1. Introduction

### 1.1. Why is Measuring Glucose Utilization so Important?

Glucose (C_6_H_12_O_6_, also known as D-glucose, dextrose, or grape sugar) is a monosaccharide and a pivotal carbohydrate in biology [1,2]. The ability to effectively monitor glucose homeostasis is crucially important in many aspects of biological research and medicine. This is because glucose is the primary source of energy in organisms and an important metabolic intermediate [1,2]. For example, glucose is a major product of photosynthesis and the starting point for cellular respiration. Moreover, high blood glucose levels in diabetes is a precursor of diabetes-associated side effects, such as blindness, atherosclerosis and neuropathy [3,4]. There is also a major need for measuring glucose flux in neuroscience. In the brain, glucose oxidation is believed to support almost all of the energy requirements of neuronal cells, via conversion to lactate by associated astrocyte cells [5]. Glucose flux is tightly linked to neuronal responses and, hence, brain activity. This has been a major reason for developing fluorescent-tagged glucose bioprobes to monitor rapid changes in glucose flux in brain tissue [5]. Contemporary research in this area has focused on defining the astrocyte-neuron metabolic relationships governing brain homeostasis and memory formation [6,7]. A further, very important reason to measure glucose flux is related to the altered metabolism of cancer cells compared to healthy cells [8]. This is known as the Warburg effect and was postulated in the 1920s by the German biochemist, Otto Warburg [9]. The rapidly proliferating cancer cells have glycolytic rates that can be up to 200 times greater than equivalent cells from their normal tissues of origin [10]. Consequently, glucose consumption is markedly increased in cancer cells and, interestingly, occurs even if oxygen is relatively plentiful. Therefore, tracking glucose uptake can delineate cancer from normal tissues, providing a means to image cancer progression and monitor response to therapeutics.

It is interesting to speculate about why glucose is the prominent fuel instead of other monosaccharides, such as fructose or galactose. One reason may be the relatively high stability of glucose compared to other monosaccharides [1]. In their open chain form, monosaccharides contain carbonyl groups that react with the amino groups of proteins to form Schiff bases, which rearrange into a relatively stable amino-ketone linkage that compromises protein function. However, the hydroxyl groups of the glucose ring are equatorial, which contributes to its relatively high stability. It should be noted, however, that glycation still occurs and is linked to complications arising from diabetes [11]. The role of glucose is also of biological importance in this context, because, the addition of glucose to proteins by enzyme-mediated glycosylation is often essential for their function [1].

### 1.2. Monitoring Glucose Utilization before the Development of Fluorescent-Tagged Glucose Bioprobes

Prior to the invention of fluorescent-tagged glucose bioprobes, radioisotopically-labeled sugars were widely used for measuring glucose utilization (*i.e.*, see [12–16]). These radiolabeled tracers include [^14^C] 2-deoxy-D-glucose [13], [^18^F] fluoro-2-deoxy-D-glucose (FDG; [17]), and [^14^C] or [^3^H]3-Omethyl-D-glucose [18,19]. It should be noted that FDG still remains an essential non-invasive tool for monitoring glucose utilization and FDG-PET is routinely used for brain mapping or cancer diagnosis and staging (*i.e.*, see [20–28]). Apart from the technical and logistical problems associated with radioactivity, these tracers cannot measure glucose uptake in viable, living cell cultures, *i.e.*, the cells have to be killed for measurement and individual cells in a diverse population cannot be monitored. In addition, 6-NBDG has been used to measure glial cell glucose transport in real-time using confocal microscopy cells [29]. Time resolution was an important consideration in this study because fluorescent techniques can resolve cellular events within seconds, whereas isotopic techniques can only resolve within minutes. This is a critical issue for cells that change their metabolic status very rapidly, which occurs in brain tissue. Subsequent glucose utilization studies using 6-NBDG and 2-NBDG compared co-cultures of glial cells and neurons [30] and cerebellar slices [31], which are also not possible with glucose isotopes. Thus, fluorescent analogues are superior to isotope analogues in terms of temporal resolution and spatial resolution. Thus, autoradiography using isotopes measure cellular phenomena in real-time; the only time point possible for analysis is not compatible with cell physiology and, therefore, should be considered as an *ex vivo* parameter. Moreover, the limits of two-photon extracellular polar-tracer (TEP), which can be used to image long-term changes in neural or glial cells in a living tissue [32,33], are a poor temporal resolution and poor spatial resolution in the millimetre range. This means that cellular phenomena can remain unresolved. An alternative method was also available for cellular studies; glucose utilization could also be monitored by light scattering effects in red blood cells, caused by volume changes after sugar transfer [34]. In addition, a volumetric method to estimate glucose transport has been utilized in tumor cells [35] and brain cells [29]. However, this method is an indirect measure of glucose transport and that changes in cell volume may affect glucose metabolism via the activation of volume-regulatory cellular mechanisms.

Therefore, the development of a fluorescent glucose bioprobe would have been advantageous, because fluorescence spectroscopy can provide a relatively sensitive platform for monitoring glucose transport [34]. This would also allow the evaluation of cell viability, which could be readily analyzed by coupling with an image analyzing system [36]. In addition, at this time there was no method to measure both glucose transport and its effect on different intracellular functions in single, viable mammalian cells or tissues.

**Figure 1.**
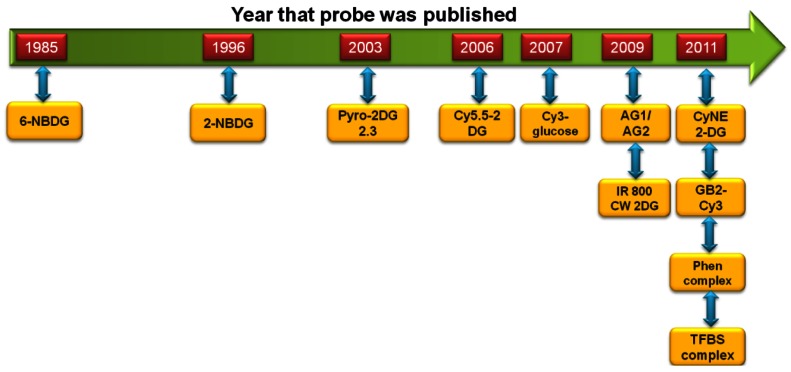
Timeline to show that research aimed at the development of fluorescent-tagged glucose bioprobes is increasing. Each new published probe is shown as a yellow box. Please refer to the relevant section of this review for a detailed explanation of each probe.

The development of fluorescent-tagged glucose bioprobes began in the 1980s [34]. However, advances in this field of research were relatively slow and it would be another 10 years before an alternative fluorescent-tagged probe would be developed, which became more widely used by the biological research community [37,38]. This spurred research in this field and in the past decade we have witnessed the development of a large number of fluorescent-tagged glucose bioprobes. These advances are shown schematically in Figure 1. Therefore, it is an appropriate time to review these advances and attempt to place them into a relevant research context, by comparing the experimental ‘strengths’ and ‘weaknesses’ of each probe.

## 2. The First Generation of Fluorescent-Tagged Glucose Bioprobes

Ideally, fluorescent tagged glucose bioprobes should possess a number of characteristics that will make them suitable as glucose analogues. These features should include a suitable molecular weight compared to glucose, low cytotoxicity, competition by glucose for cellular uptake and resistance to quenching or photo-bleaching (for further information, see [39]). In addition, the ability of the probe to be metabolized by the cell should also be considered (this aspect is addressed in the description of 2-NBDG and 6-NBDG, below).

### 2.1. Development of 6-NBDG

**Figure 2.**
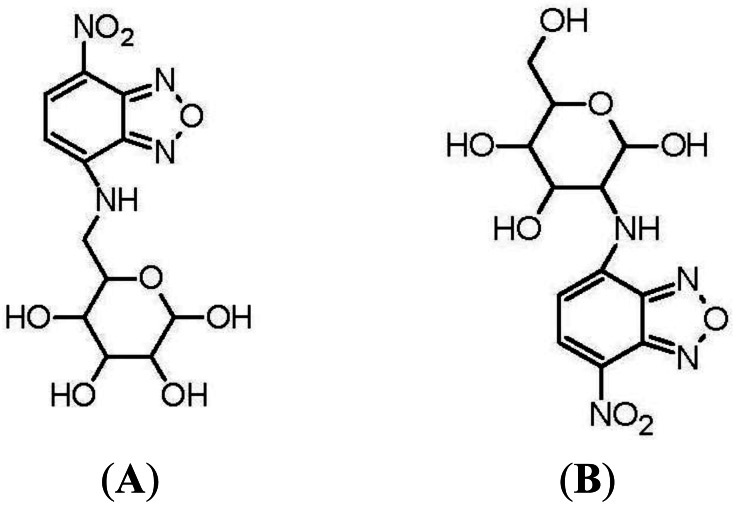
(**A**) Chemical structure of 6-deoxy-N-(7-nitrobenz-2-oxa-l,3-diazol-4-yl)-aminoglucose(6-NBDG), developed in 1985; (**B**) Chemical structure of 2-deoxy-N-(7-nitrobenz-2-oxa-l,3-diazol-4-yl)-aminoglucose(2-NBDG), developed in 1996. Both probes are commercially available from Invitrogen, Corp.

The first fluorescent-tagged glucose bioprobe was developed in 1985 [34]. The probe is 6-deoxy-N-(7-nitrobenz-2-oxa-l,3-diazol-4-yl)-aminoglucose (6-NBDG; Figure 2(A)) and it was synthesized by Professor Howard Kutchai's laboratory in the University of Virginia, in conjunction with the company Molecular Probes Inc. At that time, only one fluorescent glucose analogue had been developed: 2-deoxy-2-amino-N-(5-dimethylamino-1-naphthalene sulfonyl)-glucose (III). However, this analogue had only been used as a competitive inhibitor of hexokinase [40]. 6-NBDG was the first fluorescent glucose derivative developed to probe the behavior of glucose transport systems. In designing this fluorescent analogue, the fluorophore 7-nitrobenz-2-oxa-l,3-diazol-4-yl (NBD) was selected because of its relatively strong fluorescence [34]. The C-6 position of D-glucose was selected as the site for covalent attachment, because it was thought that the hydrophobic region of the hexose transporter is in close proximity to this part of the glucose molecule during transport into the cell [41].

Professor Kutchai and his colleagues tested 6-NBDG in red blood cells. Importantly, it was shown that 6-NBDG entry into the cells was inhibited by increasing concentrations of D-glucose [34]. This indicated that 6-NBDG entered the cell through glucose transporters (GLUTs). This was further validated by showing that entry was inhibited by cytochalasin B, which is a mycotoxin and a high affinity GLUT inhibitor that disrupts actin polymerization [42,43].

However, it was also observed that the entry rate for 6-NBDG is much slower compared to other monosaccharides. The authors speculated that this could be due to 6-NBDG having a low affinity for GLUT, or the maximal uptake (*V_max_*) for 6-NBDG uptake is much lower than glucose [34]. This point would be further addressed in an important study by Barros *et al.* in 2009 ([44] and discussed in Section 2.2).

Although 6-NBDG was made available by Molecular Probes, Inc., which was subsequently assimilated by Invitrogen, Corp., its use by the biological research community was quite sparse, because in the subsequent nine years there were only four published studies that used 6-NBDG to monitor glucose transport. These reports included assessment of the effects of phorbol esters on glucose uptake and regional differences in glucose transport in the mouse hippocampus [45]. In addition, two photon microscopy has been used to image 6-NBDG uptake in rat brain tissue *in vivo* [46]. In this study, astrocytes and neurons both take up 6-NBDG at the same rate in the barrel cortex of the rat brain. However, during intense neuronal activity triggered by whisker stimulation, rapid 6-NBDG uptake by astrocytes could be observed, whereas neuronal uptake remained almost unchanged. This latter study was important, because it demonstrated that a fluorescent-tagged glucose bioprobe could be used to monitor glucose uptake *in vivo*.

### 2.2. Development of 2-NBDG

Eleven years after the development of 6-NBDG, Hideaki Matsuoka's Laboratory, at Tokyo University of Agriculture and Technology, synthesized 2-(N-(7-nitrobenz-2-oxa-l,3-diazol-4-yl)amino)-2-deoxyglucose (2-NBDG; Figure 2(B)) [37]. Matsuoka's Research Group was interested in monitoring bacteria viability and metabolic events. The probe 6-NBDG could not be used to directly monitor cell metabolism, because the modification at the C-6 position of glucose would inhibit phosphorylation by the glycolytic enzyme, hexokinase, leading to 6-NBDG accumulation within the cells. However, NBD modification at the C-2 position of glucose, to generate 2-NBDG, would allow phosphorylation by hexokinase and subsequent degradation to non-fluorescent products. Therefore, 2-NBDG should allow the monitoring of cellular metabolic events.

Accordingly, it was found that in *Escherichia coli* 2-NBDG accumulated in living cells and not in dead cells [37]. The bacteria could be readily imaged using fluorescence microscopy with UV illumination. A further, important validation was that 2-NBDG uptake was competitively inhibited by D-glucose and not by L-glucose (an enantiomer that rarely occurs in the natural world). This suggested that the bacterial glucose transport system was involved in 2-NBDG uptake.

A rapid, follow-up study validated the intracellular fate of 2-NBDG [47]. In bacteria, 2-NBDG was shown to be phosphorylated after uptake, to produce fluorescent derivative (2-NBDG 6-phosphate), presumably by the glycolytic enzyme, hexokinase. 2-NBDG 6-phosphate was then converted back into 2-NBDG by the enzyme glucose 6-phosphatase. 2-NBDG was then rapidly degraded to non-fluorescent products by the glycolytic pathway. The cellular breakdown of 2-NBDG is a useful property, because 2-NBDG signal should indicate both the cellular glucose uptake rate and metabolic activity [47], although it should be noted that 2-NBDG may also accumulate as glycogen [48]. Indeed, the usefulness and simplicity of 2-NBDG as a tracer for eukaryote cell viability was demonstrated in the same year [49].

### 2.3. Increasing Use of 2-NBDG and 6-NBDG to Trace Glucose Transport in Different Biological Systems

Following the development of 2-NBDG, there has been an increasing number of research publications utilizing this probe. A PubMed search (U.S. National Library of Medicine National Institutes of Health) retrieved 77 publication for 2-NBDG after the year 1996, with 21 publications alone for the year 2011. Two important applications for 2-NBDG appear to account for much of this increased use. Firstly, it was shown that 2-NBDG can be used to image and measure signaling pathway-stimulated glucose uptake in single cells from the pancreas [36,38]. This was followed by a large number of publications using NBDG to validate novel anti-diabetic compounds with similar effects as the hormone insulin, *i.e.*, the promotion of glucose uptake in insulin-sensitive cells, such as adipocytes and skeletal muscle cells (*i.e.*, see [50–54]). Secondly, in cancer research, there has been increasing focus on targeting tumor cell metabolism to develop cancer drugs (reviewed in references [55–57]). This has stimulated the use of NBDG to monitor the increased glucose uptake in cancer cells (the Warburg effect, discussed in Section 1.1) [58]. For example, 2-NBDG has been used to develop high-throughput quantitative assays for glucose uptake in cancer cell lines [59] and as a method to monitor therapy response in breast cancer cell lines [60]. Importantly, potential clinical applications for 2-NBDG are being developed, for example as a tracer for cisplatin treatment in ovarian cancer [61] and as a contrast agent for detecting of Barrett's-associated neoplasia during confocal imaging [62]. A very interesting finding has been the demonstration that, *in vivo*, 2-NBDG imaging of murine tumors is possible by performing fluorescence colonoscopy [63]. In this study, NBDG-based imaging was compared and correlated with radioactive 2-deoxy-2 [18F]fluoro-D-glucose position emission tomography (FDG-PET) and it was concluded that 2-NBDG is an optical analog of FDG-PET that can extend metabolic imaging to both minimally invasive and intra-operative settings [63].

2-NBDG use has also been extended to the study of brain cells, which, compared to other cell types, rely heavily on blood glucose for their energy supply (*i.e.*, see [30,64,65]). In relation to these findings, another interesting recent report has shown that 2-NBDG can be used for localizing epileptic foci in an animal model, via injection into the tail vein [66]. Studies that show imaging of disease states in animals using 2-NBDG are important, because they can form the basis of subsequent studies which aim to replace the use of radioactive imaging agents in patients, such as FDG.

Although 6-NBDG is less popular with the biological research community than 2-NBDG (in terms of published reports), it still continues to be utilized as a glucose tracer. For example, our own research group has used insulin-stimulated 6-NBDG uptake as a test for multipotent stem cell conversion into adipocyte cells [67,68]. Interestingly, much of the published research using 6-NBDG has been physiological studies of neurons and astrocytes (*i.e.*, see [46,69,70]). In addition, 6-NBDG has been used to measure glial cell glucose transport in real-time using confocal microscopy cells [29]. Time resolution was an important consideration in this study because fluorescent techniques can resolve cellular events within seconds, whereas isotopic techniques can only resolve them within minutes. This is a critical issue for cells that change their metabolic status very rapidly, which occurs in brain tissue. Subsequent glucose utilization studies using 6-NBDG and 2-NBDG compared co-cultures of glial cells and neurons [30] and acute cerebellar slices [31], which are also not possible with glucose isotopes. Thus, fluorescent analogues are superior to isotope analogues in terms of temporal resolution and spatial resolution. The stability of 6-NBDG in cells after cellular uptake, where it accumulates instead entering the glycolytic pathway, (which is the cellular fate of 2-NBDG), is a possible reason why 6-NBDG is suitable for studying neuronal glucose transport.

Nevertheless, issues remained concerning the use of NBDG glucose probes. For example, a major issue was the kinetic behavior of these tracers, which is not conventional when compared to cellular glucose uptake. It was shown that NBDG permeates the cell 50–100 times slower than glucose [44]. Moreover, other research groups have shown that glucose cannot compete effectively with NBDG uptake [44,71]. For example, 90% inhibition of 2-deoxyglucose uptake by glucose only produces 20–40% inhibition of 6-NBDG uptake [44]. This finding appears to be confusing if one considers that NBDG enters the cell more slowly than glucose and could indicate significant GLUT-independent uptake of NBDG.

Fortunately, this issue was resolved in an important study from the laboratory of Professor Barros at the Centro de Estudios Científicos, Chile [44]. This research group found that in astrocytes, 6-NBDG binds to GLUT1 with approximately 300 times higher affinity than glucose. This finding provided a valuable, parsimonious explanation for why 6-NBDG uptake is not efficiently displaced by glucose. In addition, this group also reported that, in light of this high affinity binding and low transport through GLUT, when compared to glucose, exofacial GLUT inhibitors, such as 4,6-ethylidene-D-glucose, should be used to confirm GLUT-specific NBDG uptake (please note that cytochalasin B is an intracellular GLUT inhibitor). Although these results were obtained using 6-NBDG, the authors suggest that they can also explain the kinetics of 2-NBDG cellular uptake [44]. Further analysis of 6-NBDG uptake specificity investigated the temperature dependence of 6-NBDG uptake by astrocytes [72]. It was found that the temperature coefficient (*Q*10) of 6-NBDG uptake in astrocytes was similar to the *Q*10 of GLUT1 in erythrocytes, which was much higher than the *Q*10 for simple diffusion. This finding provides further evidence of a minor role for simple diffusion in 6-NBDG uptake.

**Figure 3.**
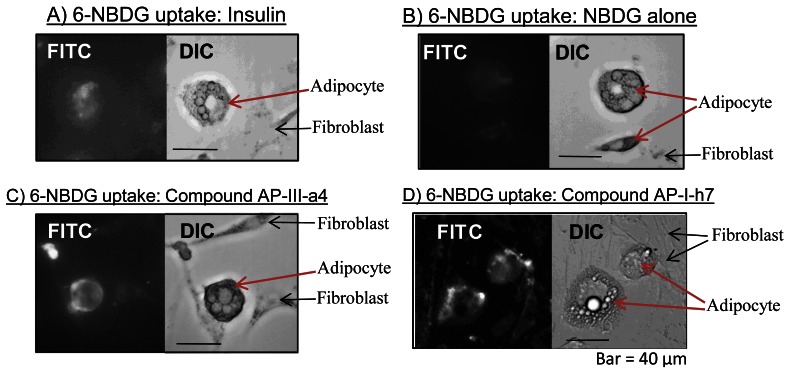
Data from our laboratory indicating that the fluorescent-tagged glucose probe, 6-NBDG, enters cells in response to insulin treatment. Two populations of cells were cultured together; fibroblasts and adipocytes, which can be distinguished under DIC microscopy by adipocyte-specific deposition of lipid droplets. (**A**) Adipocytes are insulin sensitive cells, unlike fibroblasts. In response to insulin treatment, adipocytes selectively take-up the NBDG probe (as shown by FITC fluorescence microscopy); (**B**) However, NBDG treatment without insulin produced markedly less NBDG uptake by the adipocytes; (**C,D**) Two new triazine-based insulin mimetic agents (termed AP-III-a4 and AP-I-h7) could also induce NBDG uptake selectively in adipocytes, suggesting that these compounds are attractive candidates for further development as diabetes drugs. Reference: MolBiosyst. 2011, Feb;7(2):346–358; reproduced with permission from the author).

Our own research experience with 2-NBDG and 6-NBDG has also been positive. For example, we have confirmed that NBDG is transported into cells in an insulin-dependent manner (Figure 3). Thus, the use of NBDG as a relatively fast and convenient measure of glucose transport has greatly facilitated our work, which focuses on the discovery of new diabetes drug candidates. The non-radioactive nature of NBDG probes has allowed us to develop rapid screening systems to discover compounds that induce glucose uptake, for subsequent analysis as anti-diabetic agents [53].

The proven usefulness of NBDG for diabetes, neurological and cancer research appears to be a factor in the subsequent development of alternative fluorescent-tagged glucose bioprobes by the research community. In addition, diabetes and cancer are major diseases afflicting our society [73,74]. Thus, the development superior chemical probes to study these diseases are a priority for biomedical researchers. This is especially relevant for cancer research, because the NBDG probe could not replace the radioactive glucose tracer, FDG, as a tumor imaging agent in patients. These new advances in the development of fluorescent-tagged glucose bioprobes will be discussed in the following sections of this review.

## 3. Fluorescent-Tagged Glucose Bioprobes Developed Since the Year 2000

### 3.1. Pyropheophorbide 2-deoxyglucosamide (Pyro-2DG)

**Figure 4.**
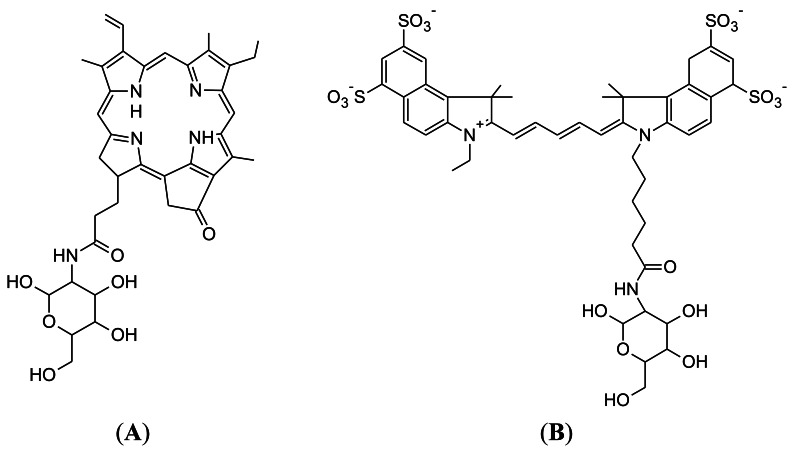
(**A**) Chemical structure of pyropheophorbide 2-deoxyglucosamide (Pyro-2DG), developed in 2003 by Gang Zheng at the University of Pennsylvania; (**B**) Chemical structure of Cy5.5-D-glucosamine (Cy5.5-2DG), developed in 2006 by Sanjiv Sam Gambhir at Stanford University.

Pyro-2DG was developed in 2003 by Gang Zheng's research group at the University of Pennsylvania (Figure 4(A)) [75]. Pyro-2DG was synthesized because 2-NBDG internalization in cancer cells had demonstrated that glucose-tagged probes larger than FDG can accumulate into tumors, in both a GLUT and hexokinase-dependent manner.

The development of Pyro-2DG marked the initiation of alternative chemical fluorescent groups for glucose attachment. Pyro-2DG contains a near-infrared (NIR) dye conjugate. NIR dyes had produced considerable research interest, because in addition to their use as a fluorescence probe for cancer detection, the NIR dye could also photo-sensitize cancer cells for treatment by photodynamic therapy (PDT) [76]. This was tested in rats that had undergone glioma tumor cell transplantation. Pyro-2DG was delivered to the tail vein of the anaesthetized rats and 30 minutes later the tumor cell graft was subjected to PDT. The tumors were then excised and imaged with a low-temperature scanning fluorometer. This confirmed that Pyro-2DG had localized to the tumors and induced noticeable tumor destruction after PDT.

This same research group rapidly published a follow-up study that assessed the effectiveness of Pyro-2DG as a fluorescent metabolic index for tumors [77]. Rat or mouse cancer models received Pyro-2DG via tail vein injection. The tumors were then surgically excised and subjected to fluorescent imaging and histopathological evaluation. In addition, flattened tumor surfaces were analyzed with a bifurcated optical fiber bundle to measure the PN/(Fp+PN) redox ratio (where PN denotes reduced pyridine nucleotides and Fp denotes oxidized flavoproteins), which is an index for measuring cell metabolism and is relatively high in tumors compared to healthy tissues. For both cancer animal models, it was found that Pyro-2DG selectively accumulated in the tumor models compared to the surrounding normal tissues at a ratio of approximately 10:1. Interestingly, there was also a high degree of correlation between the PN/(Fp+PN) redox ratio and Pyro-2DG uptake. This indicates that Pyro-2DG could be employed as an extrinsic NIR fluorescent metabolic index tracer for tumor tissue, which would allow the monitoring of metabolic responses after treatment with cancer drugs. However, in spite of these advantages, Pyro-2DG has not been utilized by the research community and there is no subsequent published report concerning this probe. This may be due to Pyro-2DG not being commercialized, which restricts its availability for researchers.

### 3.2. Cy5.5-D-glucosamine (Cy5.5-2DG)

Cy5.5-2DG was developed in by Sanjiv Sam Gambhir at Stanford University in 2006 [78]. The chemical structure of Cy5.5-2DG is shown in Figure 4(B). Like Pyro-2DG, Cy5.5-2DG is a NIR fluorescent glucose analogue synthesized for imaging tumor metabolism in living subjects. Tumor targeting of Cy5.5-2DG was assessed in two rodent subcutaneous tumor graft models and imaged using the XenogenIVIS 200 small animal fluorescent imaging system. Tumor localization was clearly visualized in living mice 30 minutes after tail vein delivery of Cy5.5-2DG. The tumor tissue:healthy tissue image contrast ratio could approach 3:1, which compares favorably with a micropositron emission tomography imaging of 4:1 for FDG [78]. However, Cy5.5-2DG uptake in tumor cells could not be inhibited by a relatively high extracellular glucose concentration, which suggests that neither GLUT or the hexokinase pathway regulate Cy5.5-2DG uptake, unlike 2-NBDG. Nevertheless, Cy5.5-2DG has greater stability in the mouse model (4 days post injection) compared to 2-NBDG. While Cy5.5-2DG can be useful in labeling cancer cells for cell trafficking and *in vivo* staining of tumors by its high specificity, it is not clear how Cy5.5-2DG localizes and is retained in tumor. This finding emphasizes the importance of fluorescent dye size when preparing fluorescent tagged-glucose analogues. Since the year 2006, there has been no published scientific report concerning this probe, despite its ability to localize in tumor tissue.

### 3.3. Cy3-Linked O-1-glycosylatedglucose (Cy3-α-glucose and Cy3-β-glucose)

**Figure 5.**
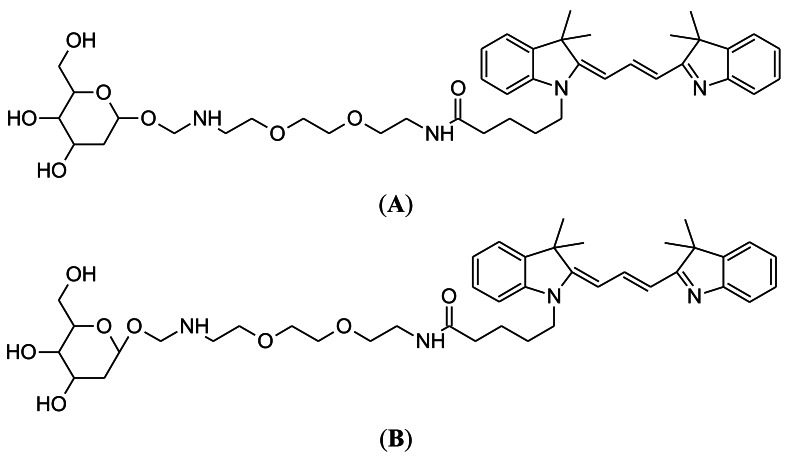
(**A,B**) Chemical structures of Cy3-linked O-1-glycosylatedglucose (Cy3-α-glucose (A) and Cy3-β-glucose (B)) developed by Park at Seoul National University.

The synthesis of both anomers (α and β) of Cy3-labeled D-glucose was reported in 2007, by the research group of Park at Seoul National University, Korea [71]. The chemical structures of Cy3-α-glucose and Cy3-β-glucose are shown in Figure 5. The Cy3 fluorescent dye was chosen for conjugation because of its compatibility with biological systems and tolerance to intense light sources, compared to the fluorescent group of 2-NBDG.

Importantly, Cy3-Glc-α labeled cancer cells more intensely than 2-NBDG (to achieve 80% of the Cy3-Glc-α signal, a 10-fold increase in 2-NBDG concentration and a 20-fold increase in fluorescence microscopy exposure time was required). Due to this relatively strong detection, Cy3-Glc-α could be applicable for bioassays without requiring glucose starvation. However, although the Cy3-Glc-α has superior imaging merits compared to 2-NBDG, it has not yet been tested in an animal tumor model. Cy3-conjugated glucose probes would be further developed in follow-up study (discussed in Section 4.2).

### 3.4. IRDye 800CW 2-DG

**Figure 6.**
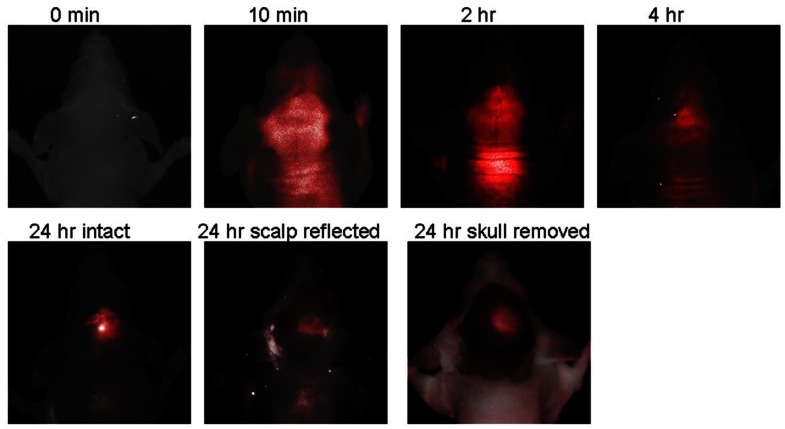
Imaging IRDye 800CW 2-DG uptake in a mouse model of intracranial glioma. For the first four hours, there was no obvious contrast between the tumor side of the brain and the normal brain. However, after 24 h later, signal remained only in the tumor side of the brain. Higher contrast was seen after scalp retraction or removal of the skull. Image reproduced from *PLoS One*; 2009, Nov 30;4(11):e8051.

IRDye 800CW 2-DG is a NIR fluorescent-conjugated glucose analogue developed in 2009 by LI-COR Biosciences, Inc. [79]. Unfortunately, to our knowledge the precise chemical structure has not been disclosed. Uptake of IRDye 800CW 2-DG in carcinoma cells was shown to be dependent on GLUT, because high extracellular glucose or a GLUT antibody inhibited probe uptake. Intravenous delivery of IRDye 800CW 2-DG in 4–6 weeks old mouse cancer models also demonstrated localization to the tumor tissue within 24 h, as visualized by the Pearl™ small animal imaging system [79]. A rapid follow-up study in 2009, from the University of Texas Southwestern Medical Center, also demonstrated the usefulness of IRDye 800CW 2-DG [80]. Probe localization to an intracranial tumor mouse model was monitored longitudinally by *in vivo* fluorescence imaging after tail vein delivery. There was significantly higher signal intensity from IRDye 800CW 2-DG in the tumor side of the brain than the contralateral hemisphere, 24 h after injection (tumor tissue/normal tissue signal ratio (TNR = 2.860.7), which was increased after removal of the scalp (TNR = 3.761.1) and skull (TNR = 4.261.1) (Figure 6). Thus, these very promising results suggest that IRDye 800CW 2-DG may serve as a useful probe for noninvasive tumor assessment in preclinical animal models. To date, these are the only published studies describing IRDye 800CW 2-DG and we eagerly await subsequent research papers describing this probe.

### 3.5. Development of Two-Photon Glucose Tracers

**Figure 7.**
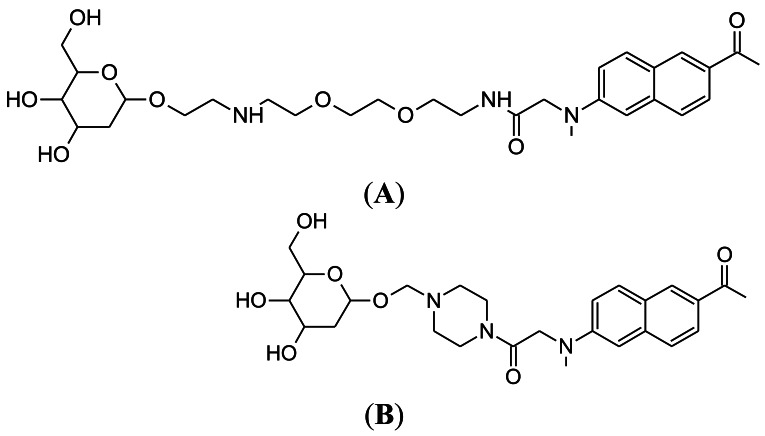
Chemical structures of the two-photon glucose analogues, AG1 (**A**) and AG2 (**B**), developed for two photon microscopy in 2009 by Park at Seoul National University.

Two-photon excitation microscopy (TPM) allows the fluorescent imaging of living tissue at a relatively high depth (up to 1 mm) [81]. Both 2-NBDG and 6-NBDG have been utilized for two photon microscopy of brain slices [31]. In addition, two photon microscopy has been used to image 6-NBDG uptake in brain tissue *in vivo* [46]. Two-photon glucose analogues were developed in 2009, from the laboratory of Park at Seoul National University (Figure 7) [82]. Development of novel TPM glucose bioprobes should address a number of parameters, including sufficient water solubility, preferential probe uptake by cancer cells due to enhanced glycolysis and a large two photon cross-section for generating a bright TPM image [82]. The chemical strategy adopted was to link glucose to acedan, which is a polarity-sensitive fluorophore that has been successfully employed in the development of TPM probes [83,84].

An important demonstration was that AG2 is taken up by cellular GLUT. In addition, AG2 uptake by tumor cells is decreased by cancer drugs, such as taxol. Moreover, TPM could produce a much brighter image for AG1 and AG2, compared to the probes 2-NBDG or Cy3-α-glucose. Additional tests showed preferential AG2 uptake in colon cancer biopsies, which was dependent on drug treatment, *i.e.*, patients receiving an anti-cancer drug showed reduced AG2 uptake in the cancer biopsy. AG2 uptake can also be monitored at a tissue depth of around 1 mm [85,86]. Thus, AG2 may be become useful in the development of customized cancer therapy for a patient, via comparison of AG2 uptake rates in healthy *versus* tumor tissue.

## 4. Recent Developments in Fluorescent Tagged Glucose Bioprobe Development (Since 2010)

Although a number of advances have been achieved in the synthesis of glucose tagged bioprobes, 2-NBDG and 6-NBDG remain the most popular choice for fluorescent-based analysis of glucose uptake in biological systems (in terms of published research papers on PubMed, US National Library of Medicine National Institutes of Health, from 2010–2011). NBDG probes remain the most popular choice because they are commercially available, relatively cheap to purchase and have been already characterized in several research publications. However, there was still no fluorescent-tagged glucose probe that could replace FDG for visualizing tumors in cancer patients. Consequently, in the past two years, a number of new fluorescent tagged glucose bioprobes have been developed. These latest research advances are discussed in the remainder of this review.

### 4.1. Development of CyNE 2-DG

**Figure 8.**
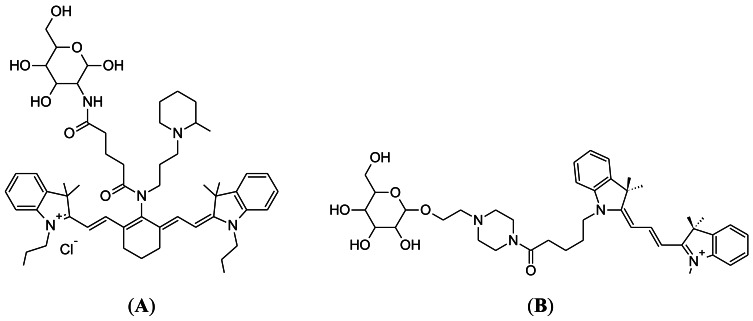
(**A**) Chemical structure of CyNE 2-DG, developed by Chang at the National University of Singapore; (**B**) Chemical structure of GB2-Cy3, developed by Park at Seoul National University.

The NIR fluorescent deoxyglucose analogue, CyNE 2-DG, was synthesized by the laboratory of Professor Young-Tae Chang at the National University of Singapore (Figure 8(A)) [87]. The performance of CyNE 2-DG was directly compared with IRDye 800CW 2-DG, with the demonstration that CyNE 2-DG produced significantly higher fluorescence signal after incubation in cancer cells. It was speculated that this difference is due to the more negatively charged chemical structure of IR Dye 800CW 2-DG, which would decrease cell membrane permeability. Importantly, high extracellular glucose could dose dependently reduce CyNE 2-DG uptake in cancer cells, indicating that cellular GLUT regulates probe uptake. However, it should be noted that tumor labeling by CyNE 2-DG was not tested in an animal cancer model.

### 4.2. Next Generation of Cy3-Labeled Glucose Probes

As a follow-up to their study in 2007, the laboratory of Park at Seoul National University synthesized seven new Cy3-labeled glucose probes [88]. The aim of this approach was to produce a Cy3-labeled glucose probe that would be applicable to the monitoring of glucose utilization in diabetes drug screening. The best probe from these seven was selected on the basis of glucose-dependent probe uptake in fibroblast cells. The probe GB2-Cy3 (Figure 8(B)) was found to produce the best fluorescent signal in the cells, which was also inhibited dose dependently by competition with extracellular glucose. In fact, this competition by extracellular glucose was greater than that measured for 2-NBDG. The performance of GB2-Cy3 was monitored in insulin-sensitive muscle cells, because novel anti-diabetic drug candidates should be able to mimic the effects of insulin [53]. GB2-Cy3 cellular uptake increased after insulin treatment and this could be blocked by wortmannin, which is a well-known insulin pathway inhibitor [89]. Importantly, GB2-Cy3 uptake can be measured in real-time using confocal laser scanning microscopy. Thus, GB2-Cy3 has the potential to become a useful glucose probe for diabetes drug screening research. This work is also of interest, because much of the other current research for glucose probe development is aimed at cancer applications, rather than diabetes.

### 4.3. Luminescent Rhenium(I) Polypyridine Glucose Conjugates

**Figure 9.**
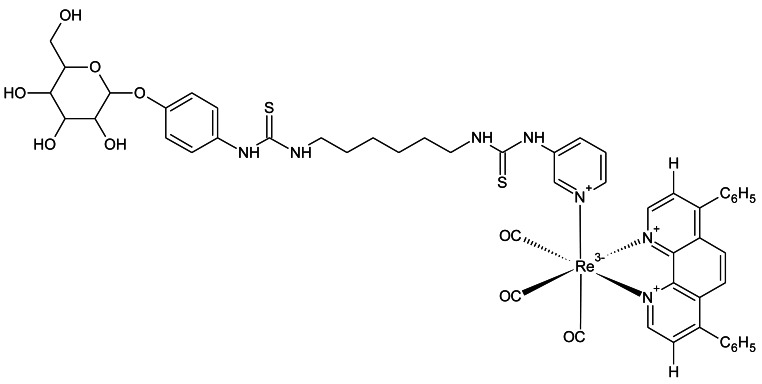
Chemical structure of the pyridine4,7-diphenyl-1,10-phenanthroline (Ph_2_-phen)rhenium(I) polypyridine glucose complex, developed by the laboratory of Professor Kenneth Kam-Wing Lo at the University of Hong Kong.

Luminescent transition-metals have also been recently investigated as tools to monitor cellular glucose uptake [90]. The laboratory of Kenneth Kam-Wing Lo, at the University of Hong Kong, has recently developed three rhenium(I) polypyridine glucose complexes and assessed their uptake in cancer cells. The chemical structure of the best performing complex (complex 3) is shown in Figure 9, as determined by both relative signal intensity and cellular uptake rates. Complex 3 performed well in cellular assays, showing uptake that was dependent on extracellular glucose concentration and decreased uptake in cancer cells treated with the cancer drug, taxol [91]. Interestingly, complex 3 was shown to accumulate in the cell mitochondria. This could also be an encouraging feature for this probe, because the glycolysis enzyme, hexokinase (that phosphorylates glucose), is localized at the mitochondria [92]. Another positive feature of the probe is photostability. Excitation for 150 seconds produced 30% decreased intensity, compared to a 90% reduction for 2-NBDG. Thus, this may become a promising probe for monitoring glucose uptake in cancer, although animal studies still need to be undertaken.

### 4.4. A New Two Photon Tracer: Star-Shaped Glycosylated Conjugated Oligomer

**Figure 10.**
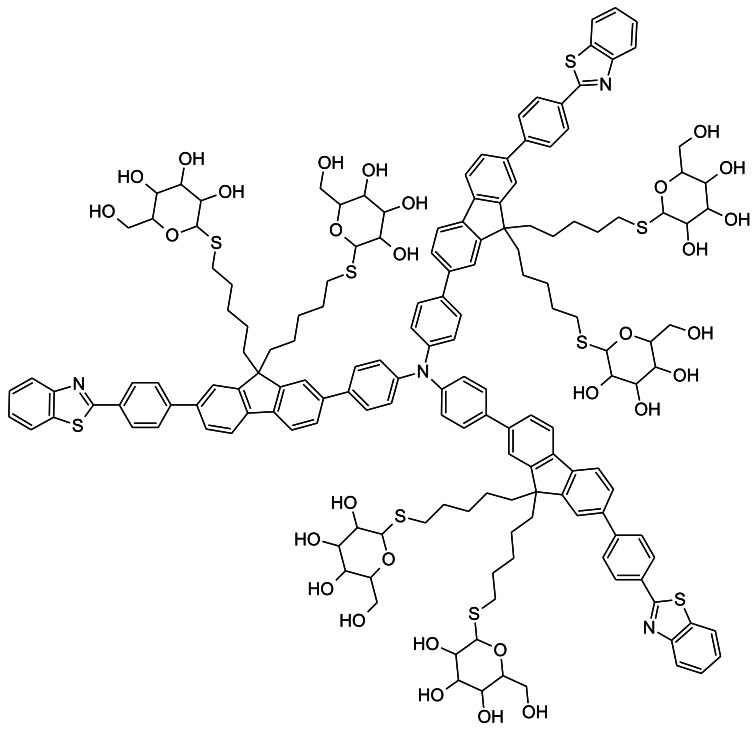
Chemical structure of the star-shaped glycosylated conjugated oligomer, fluorescent-tagged glucose bioprobe, TFBS (4,4′,4″-tris(4-(2-(4-(benzo-[d]thiazol-2-yl)phenyl)-9,9′-bis(6-thiol-β-D-glucose)-hexyl)-fluoren-7-yl)phenylamine), developed by the laboratory of Bin Liu at the National University of Singapore.

A star-shaped glycosylated conjugated oligomer, TFBS, has recently been developed by the laboratory of Bin Liu at the National University of Singapore (Figure 10) [93]. TFBS is a NIR multibranched octupolar chromophore, which was designed to maximize the cross section that can be imaged by TPM. TFBS is also water soluble, unlike other related chromophores with relatively high cross section values, making TFBS suitable for biological applications. The TFBS probe has a significantly larger molecular weight, relative to the other fluorescent-tagged glucose bioprobes described in this review. However, cellular uptake of TFBS was shown to be dependent on the presence of the glucose groups. This led the authors of this study to conclude that glucose transporters may have a role in TFBS uptake. However, further validation experiments, such as competition of cellular uptake by extracellular glucose or uptake inhibition by GLUT inhibitors, was not performed in this study. So far, TFBS has only been tested in cancer cells; staining of patient tumor biopsies was not assessed. Nevertheless, the cross sectional range for TFBS in stained cancer cells was higher than the majority of reported two-photon fluorescence probes designed for cellular imaging [93].

## 5. Conclusions

**Figure 11.**
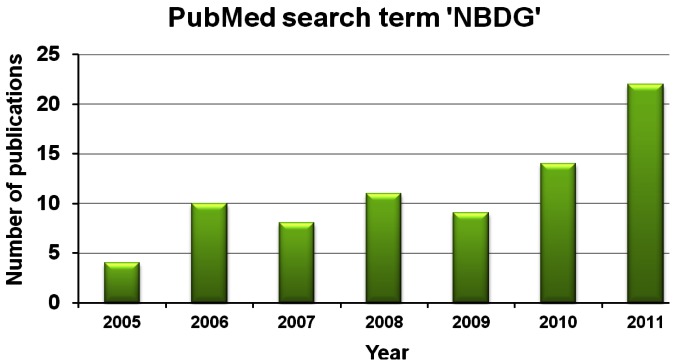
Histogram comparing research publications for 2-NBDG and 6-NBDG since the year 2005, according to PubMed (US National Library of Medicine National Institutes of Health). However, it should be noted that in 2011, there were 1,740 publications that used 2-deoxyglucose.

The development of novel fluorescent-tagged glucose bioprobes is a priority for biomedical research. Altered glucose metabolism is a fundamental aspect of cancer and diabetes [3,94]. It has been 16 years since the development of the glucose probe, 2-NBDG, but, in terms of published scientific papers per year, this probe still remains the most popular choice for laboratory researchers. Moreover, basic research publications using glucose radioisotopes, such as 2-deoxyglucose, still outnumber publications that use NBDG. However, research publications that use fluorescent-tagged glucose bioprobes are increasing each year (Figure 11), which may be due to increasing awareness among scientists that fluorescent probes can offer substantial advantages, such as superior spatial resolution for imaging and avoidance of logistical problems associated with using radioactivity in the laboratory.

This is an exciting time for the development of fluorescent-tagged glucose bioprobes, because an increasing number of novel probes are being reported each year. In addition, a number of these possess features that are superior to NBDG. Especially, new probes that utilize NIR appear to be promising for biomedical applications because they can be utilized for live imaging and are now commercially available [80]. Advances in microscopy, such as the rapid development of TPM is also being exploited by researchers [82,93] and it can be envisaged that these new TPM-optimized glucose bioprobes will soon become commercially available. Moreover, the new Cy3-labeled glucose probes also possess a number of advantages compared to NBDG [71,88]. Individual features of the currently reported fluorescent-tagged glucose bioprobes are shown in Table 1. The personal opinions of the authors of this review is that it will only be a few years before a fluorescent-tagged glucose bioprobe will be developed that can replace the current reliance on radioisotopes to visualize glucose uptake in the clinical setting.

## Figures and Tables

**Table 1.** A comparison of current fluorescent-tagged glucose bioprobes.

Probe and Year Developed	Molecular Weight	Commercial Availability	Research Application	Wavelength (nm)	Cell Types Tested	Competition with D-glucose	Tested in Animals
**6-NBDG (1985)**	342.26	Invitrogen, Corp.	Diabetes, Cancer, Neurology	Ex 470Em 538	Multiple cell types from various tissues	Yes	Mouse & Rat
**2-NBDG (1996)**	342.26	Multiple companies	Diabetes, Cancer Neurology	Ex 475Em 550	Multiple cell types from various tissues	Yes	Mammalian, fish, invertebrates
**Pyro-2DG 2.3 (2003)**	681.77	No	Cancer	Ex 665Em 720	Rat glioma	Yes	Rat
**Cy5.5-2DG (2006)**	1077.20	No	Cancer	Ex 675Em 695	Melanoma, glioma and carcinoma	No	Mouse
**Cy3-labeled α-glucose (2007)**	734.92	No	Cancer	Ex 545Em 555	Fibroblasts and carcinoma cells	Yes	No
**IRDye 800CW 2-DG (2009)**	Not published	LI-COR Biosciences	Cancer	Ex 774Em791	Fibroblasts, melanoma and carcinoma	Yes	Mouse
**AG2 (2009)**	501.57	No	Cancer	Ex 780Em 520-620	Embryonic kidney,embryonic fibroblast and carcinoma	Yes	No
**CyNE 2-DG (2011)**	970.71	No	Cancer	Ex 740Em 815	Carcinoma	Yes	No
**GB2-Cy3 (2011)**	717.91	No	Diabetes	Ex 488Em 564-606	Fibroblast and myoblast	Yes	No
**Phen complex (2011)**	1168.33	No	Cancer	Ex 405 (Em not stated)	Carcinoma	Yes	No
**TFBS (2011)**	2951.75	No	Cancer	Ex 800Em 500-600	Carcinoma	No	No
